# Spot the spy: An online game for exploring question-asking in the wild

**DOI:** 10.3758/s13428-025-02835-8

**Published:** 2025-10-21

**Authors:** Gal Sasson-Lazovsky, Vered Pnueli, Yoed N. Kenett

**Affiliations:** 1 Faculty of Data and Decision Sciences, Technion – Israel Institute of Technology, Haifa, Israel; 2 Shenkar College of Engineering and Design, Ramat-Gan, Israel

**Keywords:** Question-asking, Intelligence, Creativity, Online game, Spot the Spy

## Abstract

Question-asking is a crucial aspect of human interaction. Questions fuel engagement, stimulate thought processes, foster learning, and facilitate information-seeking behavior. Yet, scarce empirical research exists on question-asking, or its relation to related cognitive capacities such as creativity, curiosity, and intelligence. The use of digital games as a research medium offers significant advantages for studying cognitive processes in natural settings. As such, this study empirically investigates how people ask questions in an online serious game. To do so, we developed an online game—Spot the Spy—where players are required to find a hidden spy amidst a crowded room, by asking a chatbot agent questions that guide them in their investigation. Our game thus offers to investigate question-asking in natural settings empirically, and optimal question-asking strategies, which we conducted in two studies. Study 1 (online, *N* = 103) focused on game development and exploratory validation, whereas Study 2 (in-lab, *N* = 100) focused on replication and extension. In both studies, participants completed a series of cognitive tasks assessing creativity, curiosity, and intelligence before playing the game. Our results highlight strategies related to optimal performance in the game, as well as how players’ gameplay correlates with their cognitive abilities, especially with intelligence. Specifically, we found that higher intelligence scores were associated with more effective questioning strategies and better game performance. These insights highlight the potential of gamified environments to enhance our understanding of cognitive processes and advance the development of educational and training tools that foster strategic thinking and question-asking capacities.

## Introduction

Posing questions is integral to human dialogue, permeating our interactions from early childhood to adulthood (De Simone & Ruggeri, 2021; Ruggeri et al., 2016, 2019). This intrinsic aspect of conversation not only enhances social engagement but also plays a critical role in deepening mutual understanding and likability (Huang et al., 2017). The pursuit of knowledge and problem-solving endeavors is often propelled by the strategic use of questions (Gottlieb, 2021; Rothe et al., 2018). Furthermore, the selection and timing of questions are important in determining the quality and quantity of information obtained (Nelson, 2005). Yet, much is still unknown about why we ask questions, how questions vary in their quality, or how question-asking relates to various cognitive capacities, such as those facilitating information-seeking behaviors (e.g., creativity, intelligence, curiosity; Ivancovsky et al., 2024; Kenett et al., 2023; Raz & Kenett, 2024). In this study, we present an online question-asking game that we developed—Spot the Spy—to empirically study question-asking in natural settings, and relate such game performance to related cognitive capacities.

From an information-theoretic perspective, asking questions is a mechanism for reducing uncertainty (entropy) and enhancing knowledge acquisition (information gain; Gottlieb, 2021; Wang & Lake, 2019). Entropy, in this context, refers to the level of unpredictability in a set of observations, which ideally should be minimized (Crupi et al., 2018). Information gain, on the other hand, measures the value added by new data, essentially the reduction in entropy achieved by introducing new information (Coenen et al., 2019). In the context of decision-making, question-asking has been investigated particularly in terms of choosing between alternatives (Rothe et al., 2018). Through a combination of computational studies and behavioral experiments, including game-based methodologies, Rothe et al. (2018) showed how questions facilitate decision-making processes when accurately assessing question quality.

Another influential account of how people ask good questions is based on the Optimal Experiment Design (OED) framework (Boyce-Jacino & Dedeo, 2021; Coenen et al., 2019; Hawkins & Goodman, 2017; Hawkins et al., 2015). According to this framework, people choose questions to ask by aiming to maximize the knowledge they expect to gain from knowing the answer to that question (Gureckis & Markant, 2012; Myung & Pitt, 2009). However, this framework relies on multiple theoretical assumptions concerning the question askers’ prior knowledge and cognitive capacities, as well as being unsuccessful in capturing the full richness of human question-asking (Coenen et al., 2019).

In a recent theoretical review on question-asking, Raz and Kenett (2024) summarize and integrate past theories on question-asking, arguing that question-asking drives information-seeking behaviors that drive problem-solving, learning, and creativity. Thus, empirically investigating question-asking is critical across a wide range of cognitive domains.

### Empirical research of question-asking

Despite the theoretical significance of question-asking, there is a notable lack of empirical research on the topic (Raz & Kenett, 2024). This is likely due to methodological challenges in how to empirically collect, and assess, question-asking. Still, this field of research is making significant advances through the application of computational models (Wang & Lake, 2019). While such research mostly explored rule-based methods that strongly depend on a priori rules (Damassino, 2020; Gureckis & Markant, 2012), current research adapts methods from neural network models. However, such current models are still far from being able to model the full complexity of human question-asking (Wang & Lake, 2019). For example, Damassino (2020) has proposed a Questions Turing Test (QTT). In the QTT, the player needs to accomplish a yes/no inquiry in a humanlike and strategic way, i.e., with as few questions as possible. Yet, to do so, understanding the types of questions that humans ask and the process of asking questions through information seeking is necessary.

Various approaches have been proposed for how questions could be categorized and measured. By utilizing Bloom’s taxonomy (Bloom et al., 1956), questions can be assessed on a scale from one to six to reflect their cognitive level, from simple factual queries to more complex analytical or evaluative ones (Plack et al., 2007; Raz et al., 2023; Zheng et al., 2008). This framework structures different learning skills and suggests methods to gradually progress from basic knowledge acquisition to higher-order thinking skills. While the Bloom taxonomy has also received criticisms over the years (Agarwal, 2019; Larsen et al., 2022; Ormell, 1974), it can still be considered as a pragmatic operationalization of question complexity.

Another type of question classification was proposed by Mosher and Hornsby (1966), who suggested distinct types of questions based on categorical knowledge and understanding. These types of questions are aligned with different searching strategies and thus may reflect how people gather information through question construction. Constraint-seeking questions are questions that target a feature shared by multiple objects, so that they typically help to eliminate multiple options or hypotheses at each step of the searching process. Therefore, these questions are considered efficient questions compared to hypothesis-scanning questions (Ruggeri et al., 2017). Hypothesis-scanning questions reflect the use of a specific self-sufficient hypothesis and eliminate only a single option. Finally, pseudo-constraints questions are inquiries phrased like constraint-seeking questions, while they only rule out one hypothesis.

A crucial and intuitive classification of questions is into closed-ended and open-ended questions. Closed-ended and open-ended questions differ in several characteristics, especially regarding the role of respondents when answering these types of questions (Raz, Luchini, et al., 2024). Closed-ended questions limit the respondent to the set of alternative answers offered by the questions and require the respondent to home in on a single correct solution or recall a previously solved problem and apply that solution method (Jonassen, 1997). Conversely, open-ended questions allow expressing an opinion without being largely influenced by the question designer and generating multiple possible solutions (Reja et al., 2003).

Open-ended questions are information-seeking oriented and thus serve the purpose of acquiring information as they genuinely seek the truth (Çakır Sarı & Cengiz, 2016). However, research on questions reveals that closed-ended questions are used more than open-ended questions in whole-class teaching (Çakır Sarı & Cengiz, 2016). Open-ended questions are not only important tools in engaging in cognitively challenging conversations and promoting higher-order thinking, but they are also found to offer linguistic advantages as they help develop vocabulary and cognitive skills (Çakır Sarı & Cengiz, 2016). Yet only a limited understanding of the nature of question-asking currently exists (see Raz & Kenett, 2024), nor do the cognitive capacities that facilitate question-asking.

### Question-asking and cognitive capacities

What cognitive capacities drive effective question-asking? While relatively scarce empirical research exists on this topic, curiosity, intelligence, and creativity seem to be core cognitive capacities. Curiosity is theorized to be a critical motivation that drives information seeking via question-asking (Ivancovsky et al., 2024; Kedrick et al., 2023; Kenett et al., 2023; Lydon-Staley et al., 2021; Zhou et al., 2024). Furthermore, Hartung et al. (2022) have recently demonstrated how the relation between curiosity and knowledge acquisition is mediated by intelligence. The authors argue that curiosity facilitates knowledge acquisition by promoting reasoning abilities. Finally, question-asking also requires creativity (Sasson-Lazovsky et al., 2025; Torrance, 1987)—the ability to come up with original and effective ideas. Prior research has contended that the capacity for creativity is intricately linked to one’s ability to pose questions, noting a strong correlation between the types of questions asked and individual levels of creativity (Albergaria-Almeida, 2011). Their study reveals that individuals who ask primarily closed-ended questions tend to exhibit lower levels of creativity, while those who pose a variety of questions showcase higher creative aptitude (see also Acar et al., 2023; Raz et al., 2023; Raz, Luchini, et al., 2024; Raz, Reiter-Palmon, et al., 2024). Furthermore, the act of problem construction often necessitates queries about the fundamental goals of a problem-solving task, linking this process to question-asking and creative problem-solving as well (Abdulla et al., 2020; Arreola & Reiter-Palmon, 2016; Hu et al., 2010).

Another possible way to represent different types of question-asking is through the lens of creativity research, where open-ended questions are an example of divergent thinking – multiple possible solutions generated (Abdulla Alabbasi et al., 2023; Acar & Runco, 2019), while close-ended questions are termed a part of convergent thinking – convergence on one solution (Cortes et al., 2019; Guilford, 1956; Jones & Estes, 2015). Divergent and convergent thinking were historically thought to be somewhat opposing types of creative cognition (Guilford, 1956). However, Vidler (1974) found correlations between convergent and divergent thinking tests, at the individual and total level, and Smith et al. (2013) demonstrated how convergent thinking includes an initial divergent stage before participants proceed to a convergent thinking stage. Today, according to dual process models of creativity, divergent and convergent thinking are interwoven during the creative process (Allen & Thomas, 2011; Sowden et al., 2015). Specifically, divergent thinking is theorized to broaden the mental search space in which convergent thinking operates, in order to identify the best ideas for the relevant task at hand (Cortes et al., 2019). Convergent thinking might even have a threshold effect on divergent thinking, with Zhu et al. (2019) finding that only for individuals who performed better in convergent thinking tasks, divergent thinking predicted scientific creativity performance.

Thus, curiosity, intelligence, and creativity (both divergent and convergent thinking) seem to be pivotal in relation to question-asking ability. However, much of the work linking these cognitive capacities with question-asking has been indirect, and limited research has directly done so (e.g., Raz et al., 2023; Raz, Luchini, et al., 2024; Raz-Reiter-Palmon, et al., 2024). This may be due to methodological challenges in empirically studying question-asking—a context-dependent process—in standard in-lab designs. A possible solution to these challenges is to use gaming environments to study question-asking in natural settings.

### Cognitive assessment via gaming environments

Investigating question-asking poses a challenge in conventional laboratory experiments, which often fail to capture its spontaneity and real-world context. Exploring the landscape of digital games reveals a rich tapestry of question-asking dynamics, integral to both gameplay and player engagement (Pedersen et al., 2023; Rafner et al., 2022, 2023a, 2023b; Zhangozha, 2020). These platforms mirror complex aspects of the question-asking process and present an opportunity for deeper analysis and examination in more natural settings (Allen et al., 2024). More generally, there is increasing evidence for the significance of using gaming environments to study cognition, and especially high-level cognition such as creativity and intelligence (Pedersen et al., 2023; Rafner et al., 2022; Rafner, Beaty, et al., 2023; Rafner, Wang, et al., 2023).

A strong example of the feasibility and opportunities of assessing question-asking in naturalistic settings is applied research conducted in museums (Nelson et al., 2020; Su et al., 2023; Xu et al., 2024). For example, Su et al. (2023) utilize an app they developed, Dr. Discovery, to investigate museum engagement across various modes of their app, namely a simple question-asking mode and a gamified version mode. The authors collected and coded questions asked by groups during a museum visit, classifying questions across increasing levels of complexity as defined by the authors (Su et al., 2023). The authors show how the quality of questions asked in museums varies across different contexts. Importantly, the authors show how the quality of the questions asked was higher in the gamified version of the app, compared to the simple question-asking mode. This work uniquely demonstrates how the investigation of question-asking can be conducted outside of traditional classroom settings, in naturalistic settings such as museums (Su et al., 2023). However, field studies, such as those in museums, are relatively more difficult to conduct. A more fruitful direction for empirically examining human question-asking may be via online question-asking games.

A notable instance of such an online question-asking game is the Akinator, a globally popular online game where a virtual genie guesses a character the player thinks of by asking a series of questions. Research examining the Akinator’s methodology in question formulation has analyzed extensive datasets from numerous game sessions (Sasson & Kenett, 2023). This analysis revealed the potential of leveraging online question-based games to gain insights into the patterns and progression of question-asking, laying a foundational understanding for this study. However, this study was limited in scope due to the patent protection of the software. Yet, it highlighted that the Akinator’s question-asking process does not aim to narrow an information space—a popular theory on the aim of question-asking—and that the questions generated by the Akinator can be characterized into focused, yet time-evolving, topics. However, a critical limitation of this study lies in its focus on questions posed by an AI model, not by humans. This distinction is crucial as it overlooks the natural, spontaneous, and often more nuanced way humans formulate and use questions (but see Hwang et al., 2023).

The utilization of online game environments as a research medium offers a unique avenue for collecting extensive, varied data in ecologically valid settings (Allen et al., 2024; Gómez-Maureira et al., 2022; Jannai et al., 2023; Pedersen et al., 2023; Rafner et al., 2022; Rafner, Beaty, et al., 2023; Rafner, Wang, et al., 2023; Vickrey et al., 2008). These game environments authentically capture diverse player strategies and creative questioning methods, offering a richer dataset than typically available in standard research settings (Testoni et al., 2023). The variety of approaches seen in players within such serious games utilized in academic settings provides a wide spectrum of question-asking styles and strategies. This combination of real-world engagement with the convenience of data collection lays the foundation for a comprehensive analysis of question-asking mechanisms that extends beyond the confines of controlled in-lab experiments (Allen et al., 2024).

### The current study

Gamification is the process of incorporating game mechanics into various processes, programs, and platforms to create incentives and engaging experiences (Deterding et al., 2011; Gómez-Maureira et al., 2022). One genre of gamification that is particularly relevant for this research is coined “Serious Games”. Serious games are designed with the purpose of impacting the players’ real-life thoughts and behaviors in order to fulfill a purpose beyond the self-contained aim of the game (Frasca, 2007; Mitgutsch & Alvarado, 2012). Various studies have proved the effectiveness of games in creating engaging systems to attract audiences (Porat et al., 2020), yet empirical evidence about the impact of these games is rather limited (Giessen, 2015; Mitgutsch & Alvarado, 2012). Critically, there is a rapid increase in the development of ‘academic games’ that are developed as serious games in academic contexts and implemented in empirical research (Gómez-Maureira et al., 2022).

In the current study, we aim to investigate natural processes of human question-asking and explore their connections to related cognitive capacities. We introduce Spot the Spy (https://spotthespy.itch.io/spot-the-spy) – a hidden object game where players engage with the game through the process of posing questions to a chatbot that assists their gameplay. Hidden object games are a popular genre in which players must locate specific items or characters within a detailed environment (e.g., Hong et al., 2022). In Spot the Spy, players are tasked with identifying a covert spy in a room full of people, employing strategic questioning to narrow down the suspect. This process of inquiry closely resembles the well-known ‘20-questions’ game, a paradigm that has been extensively studied for its insights into question formulation and information gathering strategies (Courage, 1989; Ruggeri et al., 2016, 2019; Sasson & Kenett, 2023; Siegler, 1977; Testoni et al., 2023). In both scenarios, the player’s success hinges on their ability to ask effective, narrowing questions that progressively reduce the field of possibilities, thereby demonstrating the intricate relationship between question-asking, creativity, and problem-solving. In addition, our game is similar to the game “Guess Who?”, where players need to guess the identity of a character by posing a series of questions on their physical attributes (O’Neill, 2021). The novelty of our game, compared to other similar games, is its focus on the questions players ask and how the types of questions that they ask assist them in their gameplay. Critically, our game provides an empirical tool—as a serious game developed for academic research—which can be utilized for a wide range of empirical investigations.

Within the context of question-asking strategies, we anticipate that players will adopt a searching strategy that mirrors the Bloom’s taxonomy (1956) of question complexity. We hypothesize that players will start with broader and lower Bloom-level questions, gradually refining their inquiries as they advance through the game. This evolution from comprehensive queries to highly specific ones is envisaged as an adaptive searching strategy, facilitating players in the efficient elimination of suspects, and ultimately identifying the elusive spy. Finally, we posit that a player’s degree of creative thinking will significantly influence their question-asking behavior. Our secondary hypothesis suggests that individuals with a higher creative capacity will exhibit a more diverse spectrum of questions, spanning various cognitive levels, compared to those posed by participants with a lower creative inclination.

To assess these hypotheses, we conduct two studies examining the cognitive abilities and game performance of participants. Study 1 focused on game development and validation via an exploratory online study. Study 2 focused on game improvements as well as hypothesis-driven replication and extension of the results found in Study 1, via an in-lab study. In both studies, participants first complete a series of cognitive tasks designed to evaluate their creativity, curiosity, and intelligence. Following these assessments, the participants engage in playing the Spot the Spy game. By analyzing their performance and question-asking strategies during the game, we aim to understand how these cognitive abilities influence their approach to problem-solving and strategic inquiry. This approach provides a robust framework to explore the interplay between cognitive skills and game-based learning, offering valuable insights into the cognitive processes underlying effective question-asking and problem-solving behaviors.

## Study 1

Study 1 focused on the development of the Spot the Spy game and its validation via an exploratory online study. In addition, Study 1 serves as a proof of concept to explore the relationship between cognitive abilities and question-asking strategies within our game. Conducted online, this study aims to examine the feasibility of the game and explore how participants’ intelligence, creativity, and curiosity impact their gameplay performance and strategic inquiry. By leveraging an online platform, we were able to efficiently gather data from a diverse participant pool, providing a preliminary examination of our hypotheses in a controlled, yet scalable, environment.

### Methods

#### Participants

Sample-size estimation for Study 1 was conducted similarly to previous studies (Raz, Reiter-Palmon, et al., 2024a, 2024b), and was based on an a priori power analysis using G*Power (Version 3.1.9.7; Faul et al., 2007) for Bivariate Correlation. This analysis determined that 100 participants would be necessary to achieve a Pearson correlation effect size of .35 with 95% power, and α error probability of .05. A total of 120 participants who were recruited through Prolific Academic participated in the study for a compensation of £4. All participants were native English speakers from the United States and the United Kingdom. Seventeen participants who did not adhere to the game’s instructions, e.g., who did not ask any questions or asked questions unrelated to the subject, were excluded from the analysis. Consequently, the final analyzed sample consisted of 103 participants (50.5% female, 49.5% male;${M}_{\text{age}}$ = 36.8 years, SD = 12.3 years; mean years of formal education = 15.8 years, SD = 4.5 years). The study received ethical approval from the Technion Institutional Review Board (IRB number 2023–071), and all participants provided informed consent prior to their inclusion.

#### Spot the Spy – game design

**Fig. 1:**
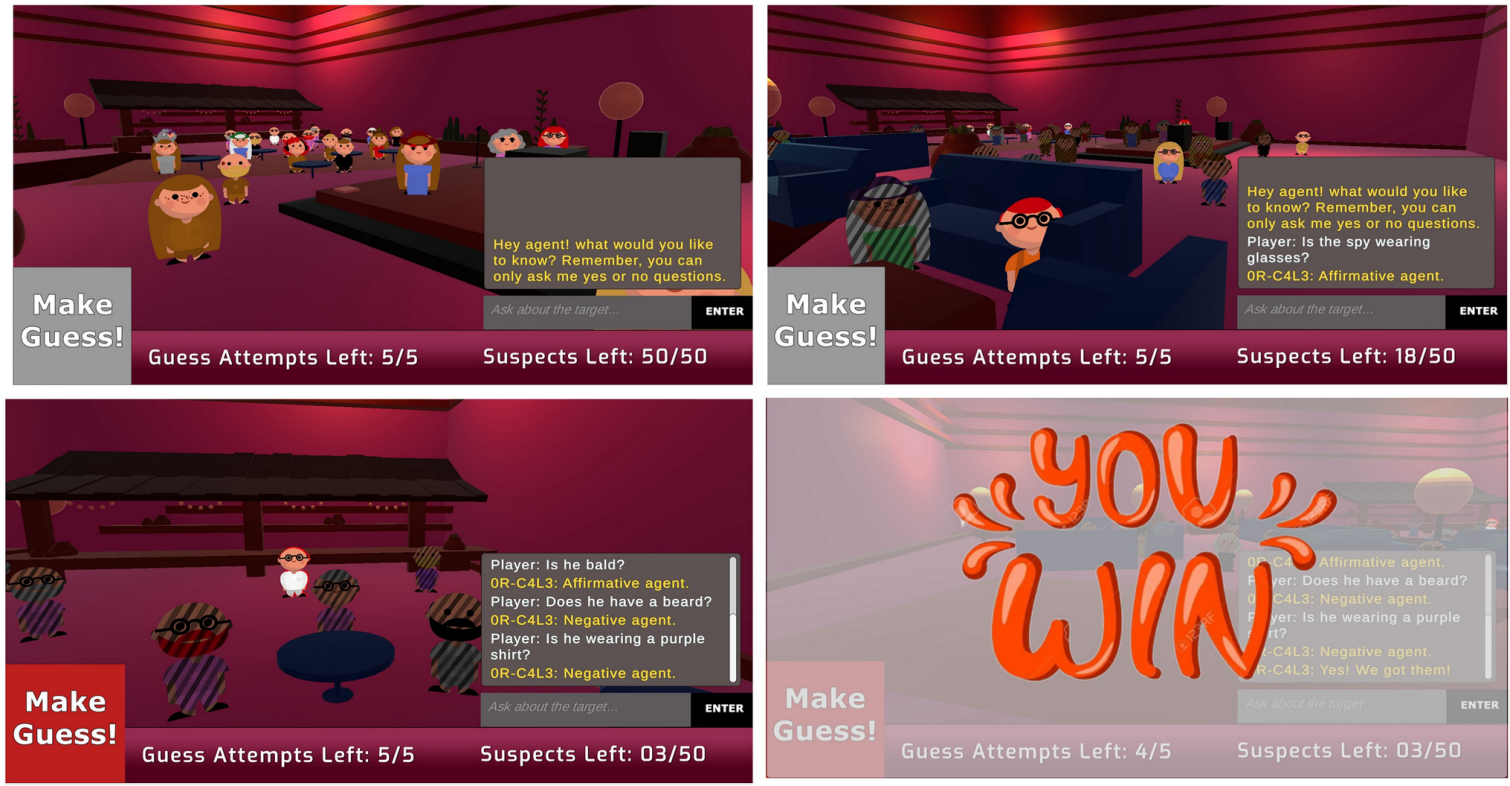
Spot the Spy game layout. (*Top left*) Opening screen of the game. The player sees a room full of suspects and can wander around the room to explore them. *(Top right*) The player asks questions about the spy to eliminate suspects. Right-clicking on suspects blacks them out to show they are eliminated. In this scene, the player finds out the spy is wearing glasses and thus eliminates all the suspects who are not wearing glasses. (*Bottom left*) The player double-clicks a suspect to make a guess. Clicking the "Make Guess!" button reveals if this is the spy. (*Bottom right*) When the player makes a correct guess, a winning screen is displayed

**Fig. 2:**
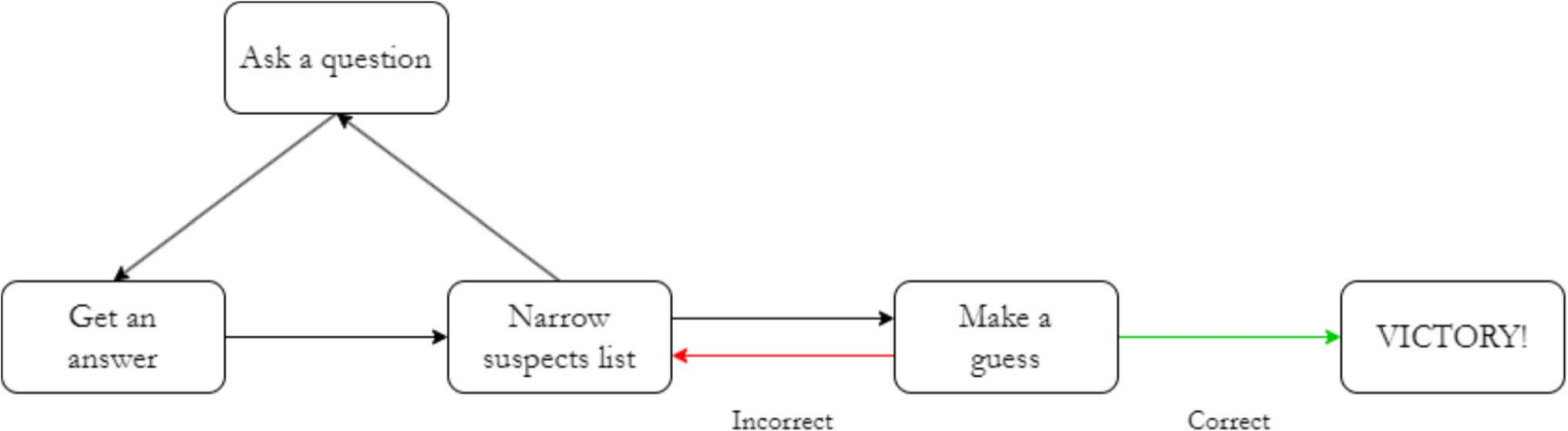
The game loop of Spot the Spy. Players pose questions to progressively refine the list of suspects before making a final guess. Correct guesses conclude the game, while incorrect ones permit further question-asking to continue

The game starts with a scene of a room full of characters who differ from each other by a set of visual features such as gender, garments, hairstyles, and accessories (Fig. 1). One of the characters is randomly selected at the beginning of the game as the “spy”, and until otherwise known, all characters in the room are considered suspects. The player can explore the different characters while wandering around the room and zooming in on various parts. Then, the player can ask a chatbot agent yes or no questions about the target (i.e., “Does the spy have gray hair?”), while visually eliminating suspects based on the answers. When the player arrives at a guess, s/he can choose a character and click the “Guess” button to get an indication of whether the guess is correct. If the player is right, the game ends; otherwise, the player can keep asking questions and making up to 5 guesses, in their attempt to identify the spy (Fig. 2).

An integral component of the game is the interactive chatbot agent, powered by GPT-4o. The chatbot assists players by providing responses to their questions, based on a pre-set list of details about the suspects. The chatbot is prompted to only respond with ‘Affirmative agent,’ ‘Negative agent,’ or ‘I cannot give the answer to that question’ in cases where a yes or no answer isn’t applicable. The instructions provided to the chatbot ensure that it answers strictly according to the predefined characteristics of the target, such as accessories, clothing, and other attributes. Additionally, the chatbot is prompted to clarify when questions are directed at it personally, redirecting the focus back to the target to prevent players’ confusion. This structured prompting aims to ensure consistency in the chatbot’s responses, allowing for a controlled analysis of participants’ questioning strategies and their adaptation based on the feedback received.

The game is hosted on the web platform itch.io, ensuring convenient access for players. As they play the game, we collect their questions, answers given to these questions by the chatbot, the properties of the spy, guesses given by the player and whether they were correct, the time it took the player to ask questions, and the progress of the player after each question (e.g., how many characters he ruled out). Finally, we use a recently developed tool—based on a trained language model—to automatically score the Bloom complexity levels of questions asked during the game (Raz, Luchini, et al., 2024). The code for the game can be found at https://github.com/galsasson1/SpotTheSpyGame.

### Measures

#### Spot the Spy

##### Game performance measures

Our game design allows to code multiple aspects of participants gameplay, including: (1) The *questions* participants ask the chatbot, the order in which they ask the questions, and the answers given by the chatbot; (2) *The number of eliminated suspects* by the participant, by visually eliminating them in the game; (3) The *guesses* made by the participant in attempting to guess the spy (number of guesses and the suspects guessed by the player as the spy), the time (in seconds) until each of the guesses was made, and whether they were correct; (4) the *time* (in seconds) it took the player to ask questions; and (5) whether the player won or lost. We analyze all these measures for every participant who played the game. Importantly, our game design is flexible, easily allowing for future additions of collected data types.

##### Question Effectiveness Measure (QEM)

In addition, we introduce a new metric, the question effectiveness measure (QEM), designed to quantitatively evaluate the impact of questions posed by players in narrowing down potential suspects. The QEM considers both the reduction in the pool of suspects following each question and the strategic sequence in which these questions are asked. In developing the QEM, we align with insights from Nelson (2005), who discusses various norms for assessing question usefulness, such as Bayesian diagnosticity and information gain (see also Nelson et al., 2010). Our approach resonates with the concept of expected stepwise information gain, as outlined by Ruggeri et al. (2017), where the informativeness of questions is measured by the reduction of entropy, thereby moving from uncertainty towards certainty. Furthermore, Testoni et al. (2023) emphasize the importance of entropy reduction in effective question-asking, particularly in scenarios where the goal is to efficiently narrow down a set of possibilities, underscoring the relevance of QEM in the context of strategic questioning in gameplay.

Moreover, the impact of object categorization levels on question-asking efficiency provides additional insights into our approach (Mosher & Hornsby, 1966; Ruggeri & Feufel, 2015). This perspective is particularly relevant to our QEM’s focus on reducing the number of suspects, similar to decreasing entropy in a set of hypotheses. The QEM can be formulated as follows:

1 $$QEM\left({q}_{i}\right)=\frac{Number of Suspects Eliminated}{Total Number of Suspects}$$

2 $$QEM=\sum_{i=1}^{n}\frac{1}{i}QEM({q}_{i})$$

where ${q}_{i}$ represents the *i*-th question in the game, with the count of suspects eliminated after each question reflecting the information gained. The total number of suspects includes all suspects, amounting to a total of 50 suspects in this study. The formula for calculating the final QEM score assigns greater significance to the effectiveness of the initial questions in the sequence by applying a weight of $1/i$, where $i$ is the position of the question in the sequence, thereby underscoring the strategic value of the first few inquiries in influencing the overall QEM score.

The QEM aims to quantify the collective efficiency of a series of questions in isolating the target, in this case, identifying the spy. It reflects both the volume of suspects eliminated and the tactical arrangement of the questions. Higher QEM values signify a more impactful sequence of questions, leading to a streamlined and effective identification process.

#### Cognitive assessments

##### Alternative Uses Task (AUT)

The AUT assesses divergent thinking, a key component of creativity (Acar & Runco, 2019; Guilford, 1967). The AUT requires participants to think of as many creative, unusual, or original uses as possible for a common object within a limited time frame. In this case, participants were asked to think of uses for a broom and a belt. They had two minutes for each object and could provide up to 30 uses for each object. The objective creativity scores of participants’ responses were computed automatically using the maximum associative distance (MAD; Yu et al., 2025), by taking the maximal semantic distance between a response to the cue word—a measure shown to strongly and quantitatively capture subjective ratings of response originality. In addition, we measure AUT fluency as the average number of responses generated by the participant to the objects.

##### Alternative Questions Task (AQT)

Similar to the AUT, in the AQT participants are provided with common objects and are asked to come up with as many original and creative questions about them as they can (Raz et al., 2023). The AQT has been shown to be positively correlated with creative thinking, especially in open-ended problem solving (Raz, Luchini et al., 2024; Raz, Reiter-Palmon et al., 2024). In this study, participants were asked to think of questions about a pencil and a pillow. They had 2 min for each object and could provide up to 30 questions for each. To score the AQT responses, we compute participants' MAD and fluency scores, and a quantitative Bloom score of each response to reflect questions’ complexity (Raz, Luchini, et al., 2024).

### Intelligence assessment

Participants underwent assessment of fluid intelligence ($Gf$) and broad retrieval ability ($Gr$). To measure *Gf*, we employed a series completion task drawn from the Culture Fair Intelligence Test. This task involved identifying the next item in a sequence of three evolving images (small line drawings) over 3 min, encompassing 16 items (Cattell & Cattell, 1961/2008). The total number of correct answers forms the *Gf* score. To measure *Gr*, we implemented a category fluency task, where participants list as many animals as possible in a 2-min period, a method previously utilized by Ardila et al. (2006). This approach allowed us to gain a nuanced understanding of the participants’ cognitive capabilities in areas crucial for our study.

### Forward flow

We included the forward flow task, as conceptualized by Gray et al. (2019). In this task, participants are initially given a cue word and are instructed to spontaneously generate the first word that comes to their mind. Subsequently, they continue this process, producing a series of words where each new word is associated with the preceding one, thus forming a chain of free associations. Computational models are used to quantify participants’ breadth of associative search (Beaty et al., 2021). Critically, broader associative ability—as measured via forward flow—has been implicated as critical in creative thinking (Beaty & Kenett, 2023). Here, we used the cue words "bear" and "table”, and participants complete a sequence of ten words for each of the cue words. This task is particularly valuable in evaluating the fluidity and connectivity of thought, essential components of creative cognition.

### Curiosity

Participants completed a 22-item questionnaire designed to measure different facets of curiosity. This questionnaire was compiled from previously established questionnaires that encompass various curiosity dimensions, including social curiosity, workplace or organizational curiosity, and I/D-type curiosity, which relates to intellectual cognition, information-seeking behavior, and perceptual curiosity (Collins et al., 2004; Kashdan et al., 2020; Litman, 2008; Litman & Jimerson, 2004; Litman & Spielberger, 2003). Participants were asked to rate the extent to which they agree with curiosity statements in relation to their own experiences and tendencies. To quantify the responses, each answer was scored on a scale from -2 (complete disagreement) to +2 (complete agreement), and these scores were then summed to yield an overall curiosity score for each participant.

#### Procedure

Participants began the study by providing informed consent, followed by completing a series of tasks on a Qualtrics questionnaire, including the AUT, AQT, fluency task, forward flow task, intelligence assessment, and a curiosity questionnaire. They also provided demographic information. Attention checks were interspersed throughout the questionnaire to ensure engagement and accuracy. After completing these tasks, participants were directed to the Spot the Spy game website for one gameplay session, until either winning or losing the game.

### Results

#### Exploratory data analysis

We first conducted an exploratory data analysis on participants’ performance in the game via the varied collected game measures. In assessing the outcomes of the Spot the Spy game, 72% of participants successfully identified the spy, demonstrating a high level of proficiency in strategic thinking and problem-solving within the game’s framework. This success rate not only highlights the effective cognitive strategies employed by the majority of players but also suggests that the game’s instructions and setup were clear and well conceived, facilitating player engagement and understanding.

**Fig. 3:**
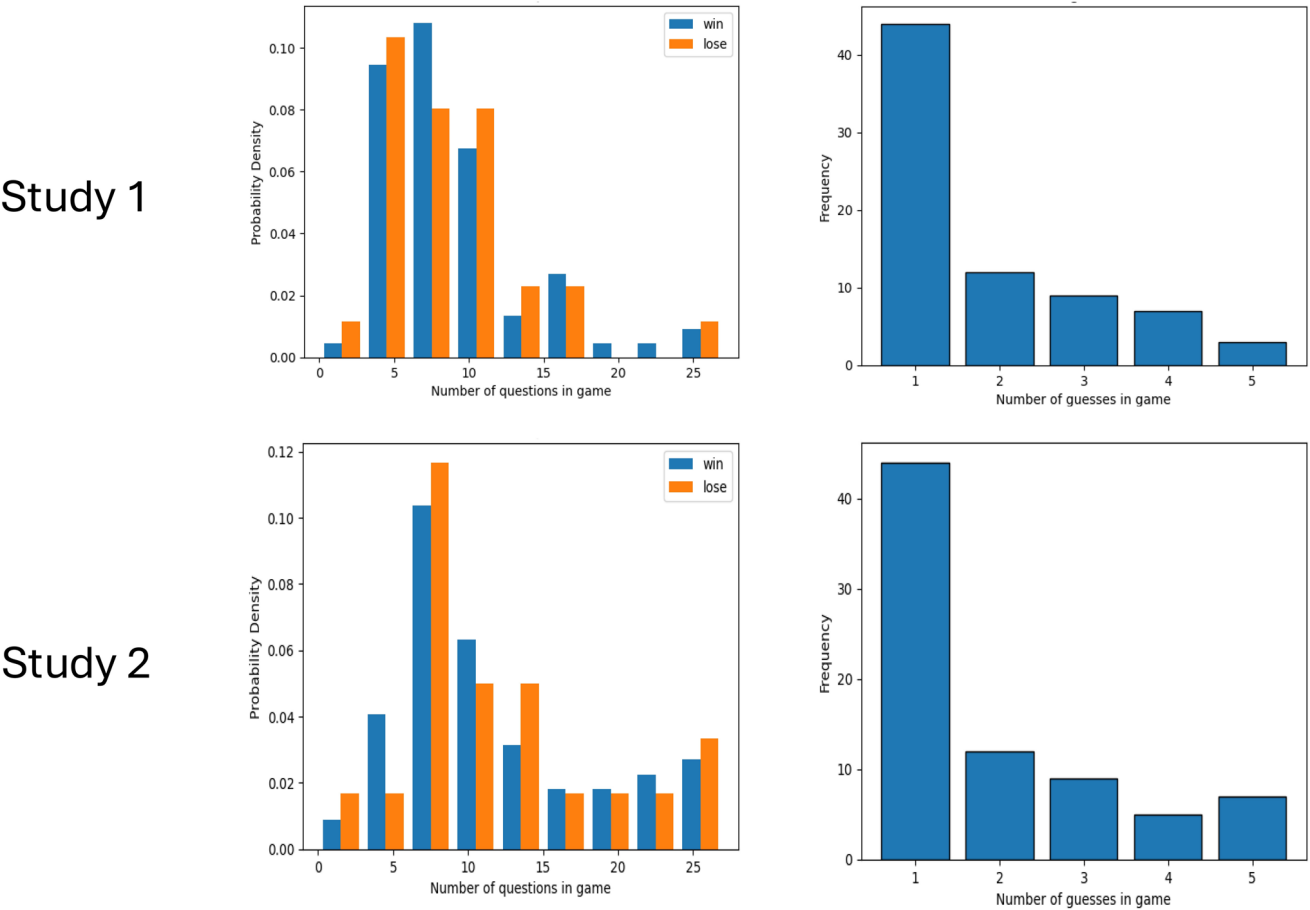
Gameplay strategies in both studies. (*Top*) Study 1; (*Bottom*) Study 2; (*Left*) The probability density distribution of the number of questions asked during a gameplay, divided by the winning status of the game. Both groups show similar distributions; (*Right*) The number of guesses of the spy taken in a game before winning. Most players win the game within one guess

Examining gameplay strategies revealed that, on average, participants posed approximately 8.71 questions (SD = 5.59) throughout their gameplay. This high standard deviation indicates a diversity in the questioning approach, with some participants asking more questions than others. Regarding the number of guesses made, among those who won the game, the average was 1.84 guesses (SD = 1.20; Fig. 3). This metric provides insight into the precision of players’ final decisions, indicating that most winners were able to accurately identify the spy with minimal guesswork. The relatively low standard deviation points to a consistent pattern of careful and calculated guessing among the successful participants.

**Fig. 4:**
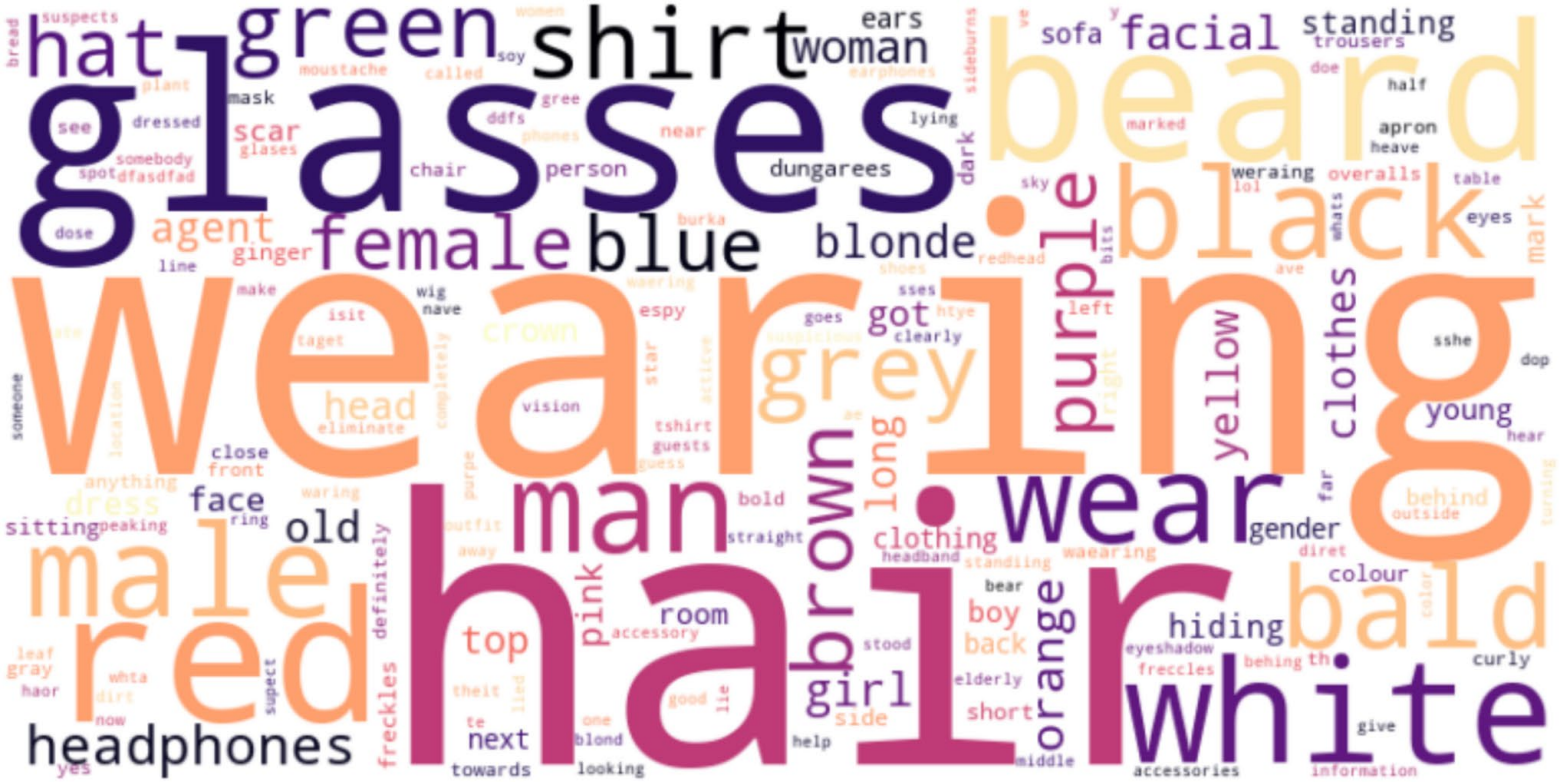
Word cloud of the most common words in questions asked by players in Study 1

Next, we examined the content of the questions asked by the players. A qualitative analysis demonstrated that the questions players asked focused on the physical attributes of the suspects (Fig. 4). We then examined the complexity of the questions players asked in the game, using the automated tool developed by Raz, Luchini, et al., (2024) based on the Bloom taxonomy (Bloom et al., 1956). These scores range from 1 (low complexity) to 6 (high complexity). In terms of the predicted Bloom complexity level of the questions in the game, the scores range between 2.21 to 4.64, with a mean score of 3.08 (SD = 0.39). The level of question complexity did not significantly change throughout the game, indicating that it is not necessary for advancement in the game or for better performance. This is likely due to the closed-ended nature of the game (Raz, Luchini, et al., 2024; Raz, Reiter-Palmon, et al., 2024).

**Fig. 5:**
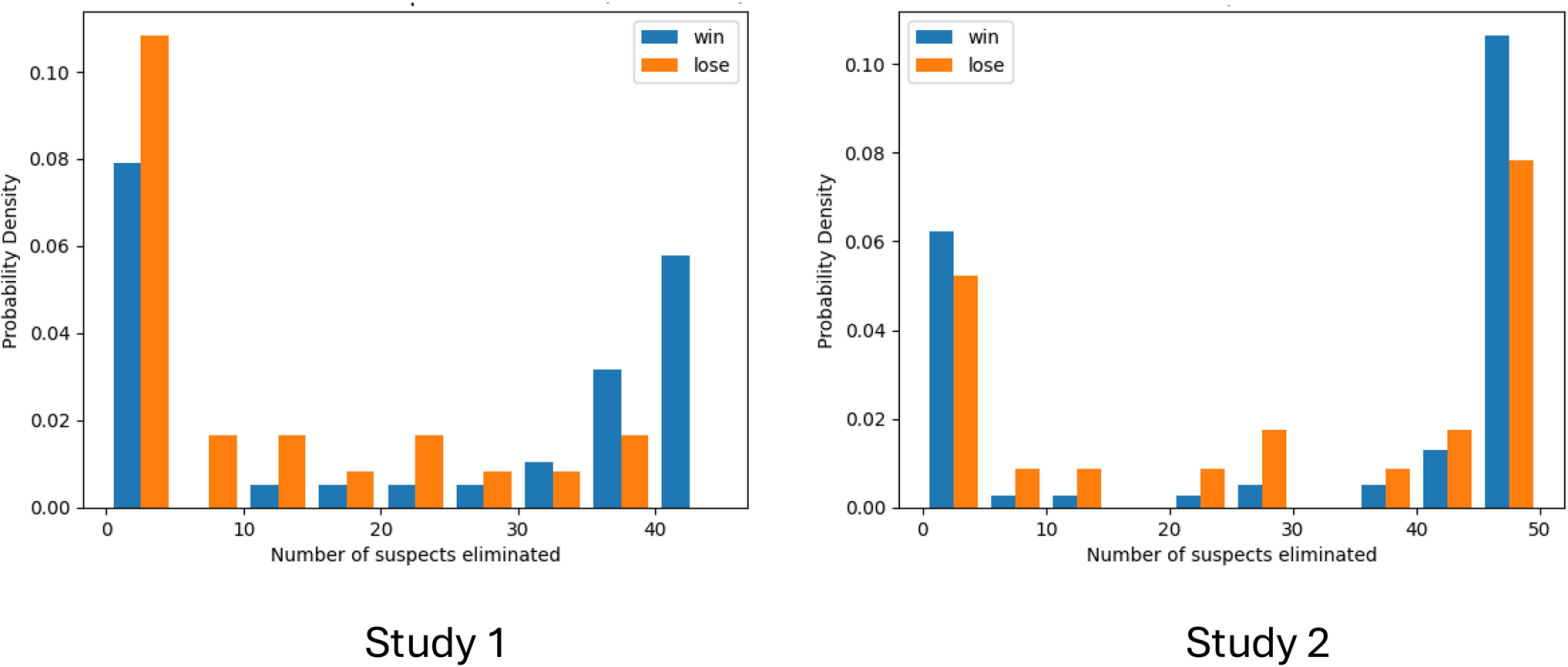
Eliminating suspects and probability of winning in both studies. The probability density distribution of the total number of suspect players eliminated during a gameplay, divided by winning status in Study 1 (*left*) and Study 2 (*right*)

A particularly interesting finding emerged when analyzing the number of suspects eliminated by participants. The average number of suspects eliminated was 30.07 suspects (SD = 19.92). However, a deeper examination of the data uncovered a distinct pattern based on the game’s outcome. Players who eliminated more than 40 suspects consistently won the game, suggesting a robust and comprehensive approach to how narrowing down suspects is linked to higher success rates. Conversely, most participants who lost the game eliminated less than ten suspects (Fig. 5). This disparity suggests that a more thorough and persistent investigative process may be key to succeeding in the game. It is unclear whether players who did not eliminate suspects chose not to use this tool or did not understand how to use it.

To further analyze this pattern, we conducted a logistic regression analysis to predict game outcomes based on the number of suspects eliminated by participants. Given the imbalance in our dataset (72% winning rate), we utilized the Synthetic Minority Over-Sampling Technique (SMOTE) to balance the class distribution (Chawla et al., 2002; Fernández et al., 2018). SMOTE is an effective approach for handling imbalanced datasets by artificially generating synthetic samples for the minority class. In our case, this technique was necessary to mitigate the bias toward the majority class (winners) and ensure a more reliable and generalizable model. After applying SMOTE, the class distribution was balanced with equal counts for both ‘win’ and ‘loss’ labels in the training set, which significantly improved the model’s predictive ability. The balanced model achieved an accuracy of approximately 95%, demonstrating its effectiveness in predicting game outcomes. In terms of precision, recall, and F1-score, the model exhibited a high precision (0.80) and perfect recall (1.00) for predicting losses, along with perfect precision (1.00) and a recall of 0.94 for wins. Precision refers to the proportion of true positive predictions (e.g., correctly predicted outcomes) out of all predictions made for a particular outcome (e.g., all predicted wins or losses), indicating how accurate the predictions are. On the other hand, recall measures the proportion of true-positive predictions out of all actual instances of that outcome (e.g., all actual wins or losses), showing how well the model captures all relevant instances. The F1-score is the harmonic mean of precision and recall, providing a single metric that balances the two, thus, it is especially useful when dealing with imbalanced datasets (Yacouby & Axman, 2020). These metrics indicate a balanced performance between precision and recall for both outcomes, with F1 scores of 0.89 for losses and 0.97 for wins. Overall, the logistic regression analysis provided deeper insights into the patterns of gameplay success, reinforcing our earlier observations about the importance of strategic questioning and problem-solving in Spot the Spy using the ability to visually eliminate suspects.

#### Correlations of study variables

**Fig. 6:**
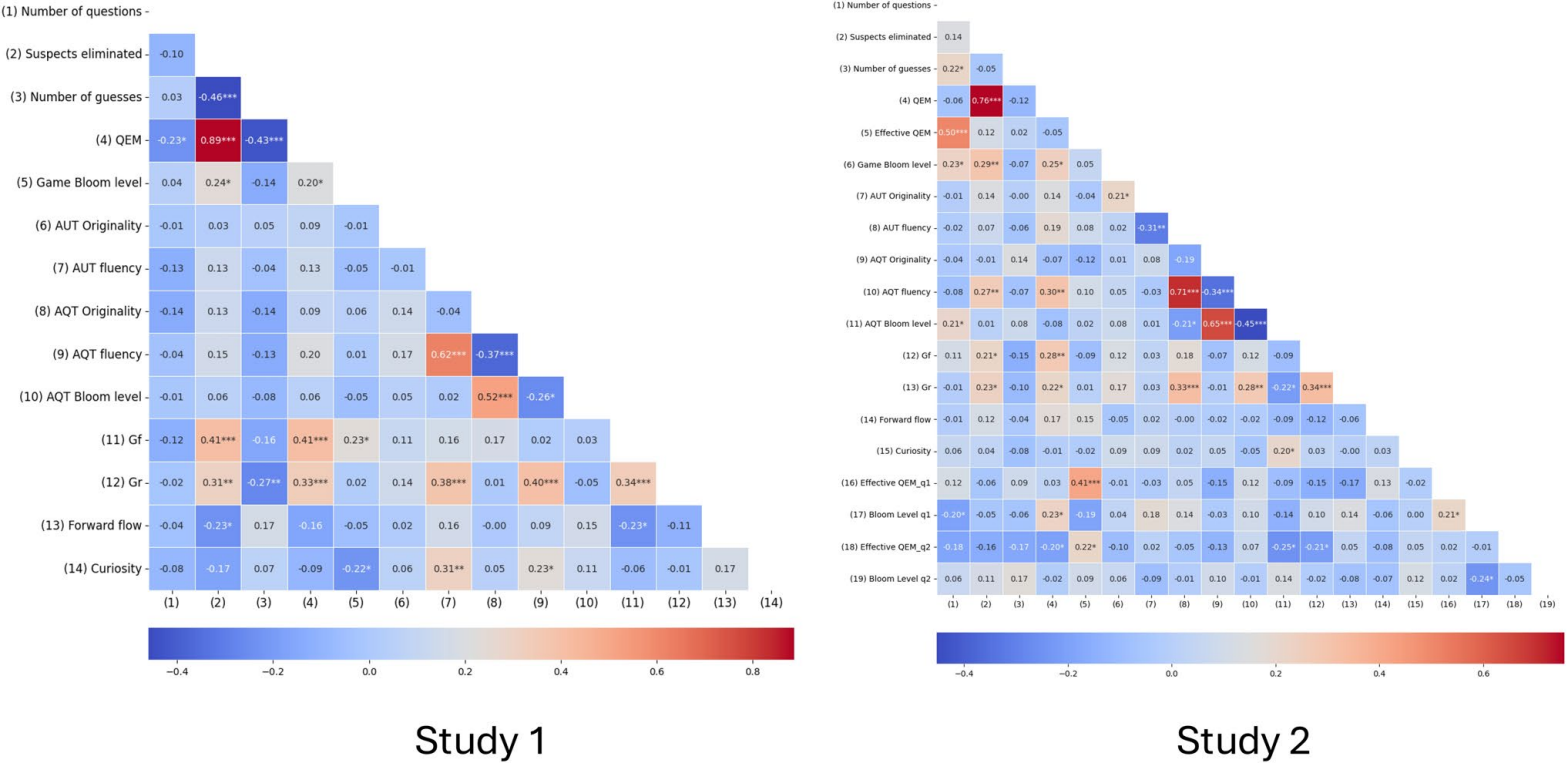
Pearson-correlations matrix between all variables of both studies. Correlations are uncorrected for multiple comparisons. *—*p* <.05, ***—*p* <.001

Next, we computed a Pearson’s correlation matrix to elucidate the interplay between variables both from the cognitive assessments and from the game performance (Fig. 6). As this study is exploratory, with a large number of variables and a relatively small sample size, we report correlation values uncorrected for multiple comparisons.

The QEM showed a significant negative correlation with the number of guesses players made in the game, *r* = –.43*, p* <.001, indicating that players who asked more effective questions tended to make fewer guesses to correctly identify the spy. This suggests that effective questioning is a key component in the game’s problem-solving process. Furthermore, the QEM showed a significant positive correlation with *Gf*, *r* =.41,* p* <.001, indicating that players with higher fluid intelligence, as facilitated in the task, tended to ask more effective questions. Additionally, there was a notable correlation between *Gf* and the number of suspects eliminated during the game, *r* =.41, *p* <.001, reinforcing the idea that higher fluid intelligence is associated with more efficient gameplay strategies.

*Gr* was also significantly correlated with the QEM, *r* =.33, *p* <.001, indicating that *Gr* contributes to the effectiveness of questioning in the game. A negative correlation was observed between *Gr* and the number of guesses, *r* = –.27, *p* =.006, suggesting that players with higher *Gr* may proceed with greater caution, requiring fewer guesses to identify the spy. There was a significant correlation between *Gr* and the number of responses in both the AUT, *r* =.38, *p* <.001, and the AQT, *r* =.40, *p* <.001. This finding indicates that participants who showed divergent thinking also tended to exhibit a similar proficiency in creating diverse responses in the AUT and AQT, pointing to a broader skill in creative ideation.

Interestingly, the curiosity score revealed a significant negative correlation with the game Bloom’s score, *r* = –.22, *p* =.023, hinting that higher curiosity might be associated with different strategic approaches, possibly reflecting more explorative behavior. Additionally, a significant positive correlation was found between the AUT fluency and the curiosity score, *r* =.31, *p* =.002, and between the AQT fluency and the curiosity score, *r* =.23, *p* =.023, indicating that individuals who are more fluent in creative tasks tend to exhibit higher levels of curiosity.

## Discussion

Study 1 focused on game development, conducting an exploratory proof-of-concept of the Spot the Spy game, and examining how it relates to various cognitive capacities that we expected to impact participants’ gameplay. A significant portion of participants successfully identified the spy, indicating effective engagement with the game’s challenges. The correlation analyses revealed that higher fluid intelligence (*Gf*) and broad retrieval ability (*Gr*) were strongly linked to more effective questioning strategies, leading to successful outcomes.

Moreover, the findings showed that a thorough elimination of suspects was crucial for success, with participants who eliminated more than 40 suspects consistently winning the game. This underscores the importance of a comprehensive and methodical approach to problem-solving. The logistic regression analysis further reinforced these insights, demonstrating that strategic questioning and the ability to visually eliminate suspects were key predictors of game success.

## Study 2

In Study 2, we aim to replicate the findings from Study 1 and further investigate the relationship between cognitive abilities and question-asking strategies within the Spot the Spy game. While Study 1 provided initial evidence and served as a proof of concept, a follow-up study is necessary to confirm these results and ensure their robustness. Thus, Study 2 is conducted within the same framework as Study 1, with minor improvements to the game to address some of the limitations that arose previously. This second study aims to examine the consistency of the observed patterns found in Study 1 and provide a deeper understanding of how intelligence, creativity, and curiosity influence gameplay performance and strategic thinking and inquiry.

### Methods

#### Participants

The sample size in Study 2 was determined similarly to Study 1. A total of 119 university students recruited from the Technion – Israel Institute of Technology, participated in the study for a compensation of 20 NIS or 0.5 credit points in academic courses. All participants were English speakers from Israel. Eighteen participants who did not adhere to the game’s instructions were excluded from the analysis. An additional participant was excluded due to their performance being more than 3 standard deviations from the sample. Consequently, the final analyzed sample consisted of 100 participants (44% female, 56% male;${M}_{\text{age}}$ = 24.5 years, SD = 3.8 years; mean years of formal education = 14.7 years, SD = 1.6 years). The study received ethical approval from the Technion Institutional Review Board (IRB number 2023–071), and all participants provided informed consent prior to their inclusion.

#### Spot the Spy - game design

The game design of Spot the Spy was identical to the one used in Study 1. Additionally, the prompting of the chatbot was improved to provide more accurate answers. For example, we specifically instructed the chatbot not to provide answers about itself, e.g., for a question such as “Are you wearing glasses?” the chatbot was asked to respond with “I am not the spy, so that’s not relevant. What do you want to know about the spy?”

### Measures

#### Spot the Spy 

##### Game performance measures

In addition to saving the questions, answers given to these questions by the chatbot, guesses given by the player and whether they were correct, the time it took the player to ask questions, and the progress of the player after each question (as in Study 1), we also saved the details of all the 50 suspects in the scene, including their properties and their location on the screen.

##### Effective QEM

For this study, we kept the entire list of the suspects and their properties in the game. Thus, the QEM can now take into account the potential number of suspects that could be eliminated following the answer given to the question. That is, we compute the effective QEM based on the ground truth without considering the players’ actions in eliminating suspects in the room. The new formulation is as follows:

3 $$effective\_QEM\left({q}_{i}\right)=\frac{Number of Potential Suspects Eliminated}{Total Number of Suspects Left}$$

4 $$effective\_QEM=\sum_{i=1}^{n}\frac{1}{i}QEM({q}_{i})$$

To compute the number of potential suspects eliminated, we first classified each question based on predefined properties and attributes. We defined mappings for properties and their corresponding keywords, such as gender (e.g., man, woman, boy, girl), hair length (e.g., bald, short), face accessories (e.g., glasses, scar), clothes (e.g., t-shirt, dress), and colors (e.g., black, red). We also included specific property-color pairs to more accurately classify questions about colors, such as hair color or clothing color. Then, each question was classified based on the presence of keywords or their associated properties, a technique called “pattern matching”. For example, the question “Is it a boy?” was classified as a question about gender due to the presence of the word “boy”. If the question included a color, we checked the property-color pairs to accurately classify the question. Thus, the question “Does the target have grey hair?” was classified as a question about hair color due to the presence of the color word “grey” and the word “hair”. The answer given by the chatbot (affirmative or negative) was then used to determine the number of suspects potentially eliminated. For each classified question, we counted the number of suspects that matched or did not match the given attribute based on the answer provided. Therefore, we get the number of potential suspects eliminated, and can reduce this number from the number of suspects that are left in the next question.

#### Cognitive assessment

Identical to Study 1.

#### Procedure

The procedure was identical to Study 1.

### Results

#### Exploratory data analysis

Similar to Study 1, we first conducted exploratory data analysis on participants' performance in the game. In Study 2, 77% of the participants successfully identified the spy in the Spot the Spy game, indicating a continued high level of proficiency in strategic thinking and problem-solving within the game’s framework. The consistent performance across both studies suggests a robust engagement and understanding among players, highlighting the game’s ability to stimulate and challenge strategic thinking skills. Participants posed an average of 12.36 questions (SD = 7.94) during gameplay, indicating a more varied and exploratory approach to gathering information compared to Study 1. The higher standard deviation of the number of questions asked in Study 2 compared to Study 1 suggests a wider range of questioning behaviors among players. Additionally, the average number of guesses made by those who won the game in Study 2 was 1.95 (SD = 1.34), slightly higher than in Study 1 (Fig. 3). These findings suggest that, while players in Study 2 tended to ask more questions and make slightly more guesses compared to Study 1, they were still able to effectively strategize and make informed decisions to identify the spy.

As in Study 1, players who eliminated a large number of suspects (close to the maximum possible of 50) tended to win, underscoring the importance of a comprehensive and exhaustive elimination strategy (Fig. 5). Interestingly, a subset of winning participants eliminated very few suspects, suggesting that there might be alternative successful strategies or that some players might have leveraged other game aspects effectively, such as early correct guesses or avoiding unnecessary eliminations. Conversely, many losing participants fell into two distinct groups: those who eliminated very few suspects and those who eliminated many suspects but still lost (Fig. 5). The former group might indicate players who did not engage fully with the game’s elimination tool, either due to a lack of understanding or different strategic choices. The latter group suggests that while a high number of eliminations is generally associated with winning, it is not a guaranteed path to success.

#### Correlations of study variables

Similar to Study 1, we computed a Pearson’s correlation matrix to examine the relationships between the variables from both the cognitive assessments and game performance (similarly reporting uncorrected correlations; Fig. 6). The number of questions asked by players showed a significant positive correlation with the number of guesses they made in the game, *r* =.22, *p* = 0.03, suggesting that players who asked more questions tended to make more guesses to correctly identify the spy, possibly indicating non-effective questioning.

The number of suspects eliminated during the game showed a significant positive relationship with *Gf*, *r* =.21, *p* =.031, as in Study 1, and with *Gr*, *r* =.23, *p* =.019. These findings suggest that both aspects of intelligence—fluid intelligence and broad retrieval ability—as captured by the tasks, play a crucial role in enhancing problem-solving efficiency, as evidenced by the higher number of suspects eliminated during the game. Additionally, the number of suspects eliminated was significantly correlated with AQT fluency, *r* =.27, *p* =.008.

Several correlations were observed with the game Bloom’s level. There was a significant positive correlation between the game Bloom’s level and the number of questions asked in the game, *r* =.23, *p* =.022, which might imply that more complex questions are not necessarily helpful in advancing in this game, requiring players to ask more questions. As in Study 1, the game questions Bloom’s level correlated with the number of suspects eliminated, *r* =.29, *p* =.003, either indicating that complex questions eventually help eliminate more suspects or that players who tend to ask more complex questions also tend to choose an effective gaming strategy more often. Furthermore, the game questions Bloom’s level showed a significant positive correlation with AUT originality, *r* =.21, *p* =.035, indicating a connection between creativity and strategic questioning in the game.

The first question’s Bloom’s level had a marginally significant negative correlation with the number of questions asked, *r* = –.20, *p* =.046, and a positive correlation with the first question’s effective QEM, *r* =.21, *p* =.04. This suggests that more complex initial questions can help in eliminating more suspects and lead to fewer overall questions being asked later in the game. Additionally, a significant negative correlation was found between the second question’s Blooms’ level and the first question’s Bloom’s level, *r* = –.24, *p* =.018, indicating that more complex first questions tend to affect the overall questioning strategy, in this case by being followed by less complex second questions.

Overall, the correlations in Study 2 partially replicate results from Study 1, highlighting the importance of intelligence and fluency in effective gameplay and creative tasks. These results emphasize the interconnectedness of cognitive abilities, creativity, and problem-solving effectiveness.

#### Analysis by intelligence level

**Fig. 7:**
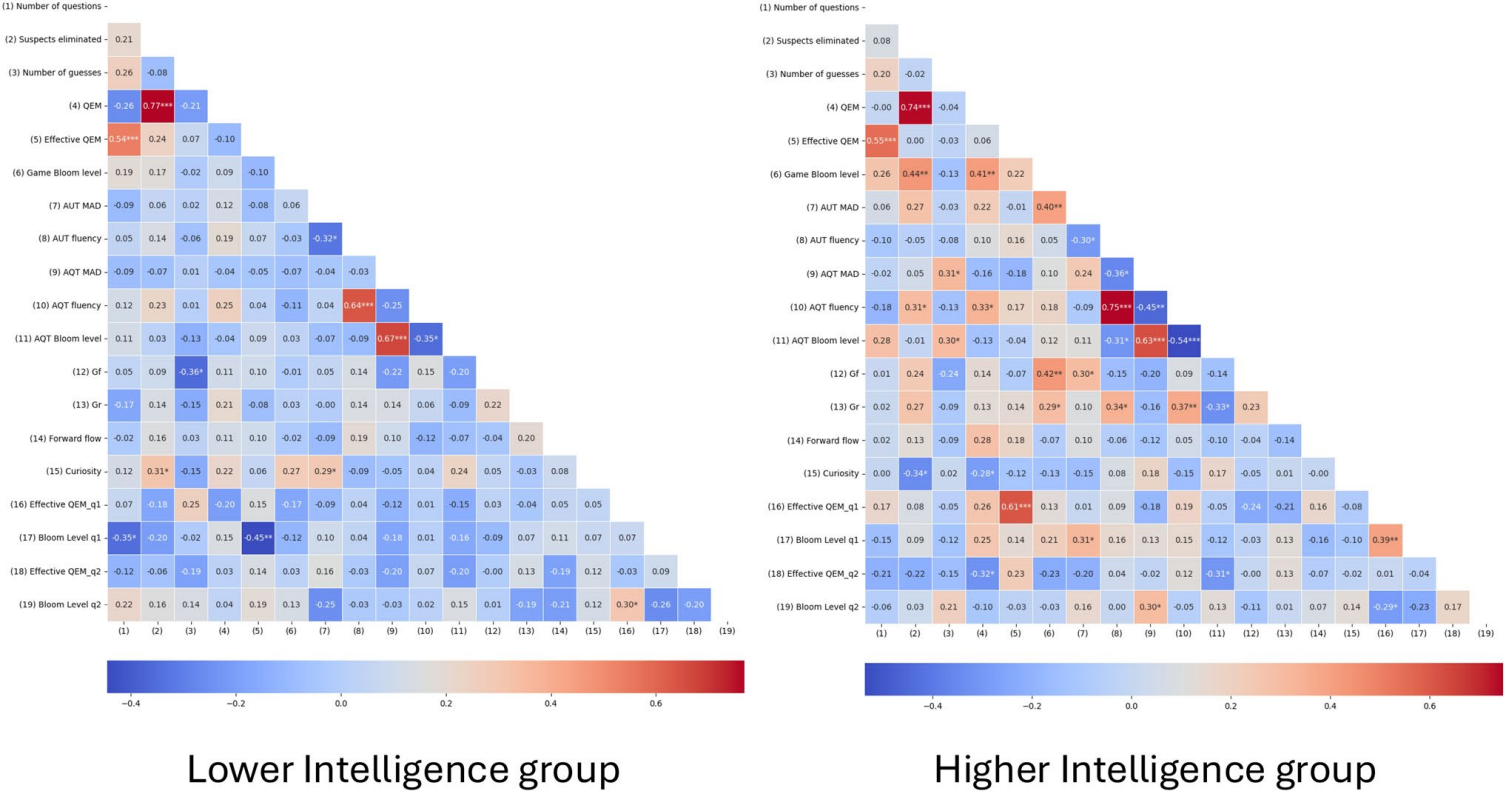
Pearson correlation matrix between all variables of the lower (*left*) and higher (*right*) intelligence groups. Correlations are uncorrected for multiple comparisons. *—*p* <.05, **—*p* <.01, ***—*p* <.001

To further analyze the correlation patterns, we divided participants into two groups based on their intelligence (*Gf*) scores according to a median split representing low and high intelligence groups, i.e., below or above the median intelligence score, relatively (median *Gf* = 8, participants with the median score were not included in this analysis). This exploratory analysis was conducted based on the relations found between performance in the game and intelligence in both studies. The low intelligence group consisted of 43 participants with a mean intelligence score of 6.28 (SD = 0.85), while the high intelligence group included 37 participants with a mean intelligence score of 9.57 (SD = 0.73). The difference in intelligence scores between the two groups was statistically significant, *t*(18.59), *p* <.001. By examining the data through the distinction of intelligence levels, we aim to see how varying degrees of cognitive capability influence the relationships between the study variables, thereby providing deeper insights into the underlying mechanisms driving performance and creativity in the game (Fig. 7).

In the low intelligence group, we find several noticeable correlations. There was a significant negative correlation between *Gf* and the number of guesses, *r* = –.37, *p* =.015, suggesting that participants with lower fluid intelligence tended to make more guesses to identify the spy. The first question’s Bloom’s score had a significant negative correlation with the number of questions asked, *r* = –.40, *p* =.01, suggesting that more complex initial questions led to fewer questions throughout the game, contrary to what we observed in the overall analysis.

In the high intelligence group, different patterns were found. A significant positive correlation was found between *Gf* and the game Bloom’s level, *r* =.43, *p* =.007, suggesting that higher fluid intelligence was linked to more complex questions in the game. Similarly, there was a positive correlation between *Gr* and the game Bloom’s level, *r* =.38, *p* =.02, indicating that higher retrieval ability was also associated with more complex questions. In addition, there was a significant negative correlation between curiosity and the number of suspects eliminated, *r* = –.43, *p* =.007, suggesting that more curious participants were less effective in eliminating suspects. Finally, the first question’s Bloom’s score was positively correlated with the first question’s effective QEM, *r* =.39, *p* =.048, indicating that more complex first questions led to a more effective questions, as seen in the overall results.

### Discussion

The findings of Study 2 replicate and extend the findings of Study 1, emphasizing the role of cognitive abilities in strategic questioning and problem-solving within the Spot the Spy game. With a 77% success rate in identifying the spy, participants demonstrated a high level of proficiency, similar to Study 1. The study revealed that higher fluid intelligence (*Gf*) and broad retrieval ability (*Gr*) were associated with more suspects eliminated, emphasizing their importance in effective gameplay strategies.

Dividing participants by intelligence levels highlighted distinct patterns: lower intelligence participants with higher curiosity and complex initial questions asked fewer questions but were less effective, while higher intelligence participants showed strong connections between intelligence, complex questioning, and problem-solving efficiency. These results highlight the intricate relationship between cognitive abilities and strategic decision-making in the game. However, it is important to note that the participants in this study were all higher-education students, representing a different population than the participants in Study 1. Additionally, they were not native English speakers, which may have affected their question-asking strategies and overall performance in the tasks and the game.

## General discussion

The practice of question-asking, an essential component of human interactions, plays a pivotal role in fostering engagement, stimulating thought processes, and driving curiosity. Its importance spans over various domains, from educational settings to everyday decision-making, emphasizing its relevance in understanding human cognition and creativity (Raz & Kenett, 2024). The current study delves deeper into the dynamics of question-asking, particularly in the context of online gaming, which mimics real-life scenarios by requiring strategic inquiry and problem-solving.

We develop and introduce the Spot the Spy game, where participants engage in a hidden object game necessitating strategic questioning to identify a covert spy among many characters. Our academic game—games developed for academic purposes (Gómez-Maureira et al., 2022)—provides a unique empirical opportunity to collect, quantify, and analyze the types and effectiveness of questions posed by players, compared to similar commercial games (Sasson & Kenett, 2023). In Study 1, we focus on the development of the game and its validation via an exploratory online study. In Study 2, we replicate and extend the findings of Study 1, via a hypothesis-driven in-lab study. In both studies, participants play the game, as well as undergo assessment of related cognitive capacities such as intelligence, creativity, and curiosity. Such an exploratory proof of concept allowed us to not only investigate how people play our game but also examine the relationship between question-asking in natural settings to cognitive capacities that relate to information-seeking behaviors, such as creativity, curiosity, and intelligence (Ivancovsky et al., 2024; Kenett et al., 2023; Raz & Kenett, 2024).

### Investigating question-asking performance in Spot the Spy

The use of digital games as a research medium offers significant advantages for studying cognitive processes in natural settings (Allen et al., 2024; Su et al., 2023). Games like Spot the Spy provide a controlled yet engaging environment where participants’ question-asking strategies and problem-solving skills can be observed and analyzed in real-time. Such an academic game approach (Gómez-Maureira et al., 2022) allows for the collection of rich, ecologically valid data, capturing the spontaneity and complexity of human cognition more effectively than in traditional laboratory settings (Allen et al., 2024; Pedersen et al., 2023). Moreover, it enables the creation of large datasets, leveraging big data to uncover patterns and insights that may not be apparent in smaller-scale studies.

Exploratory data analysis of participants’ gameplay across both studies revealed that the majority of players win the game (over 70% in each of the studies). This success rate suggests that a moderate difficulty level—where success is frequent but not guaranteed—supports variability in game performance as well as facilitates engagement in the game, as highlighted by cognitive load theory (Sweller, 1988). Furthermore, game design theory supports such a medium-level difficulty, as games that are more difficult to win challenge players and increase their enjoyment of the game (Koster, 2013). Finally, empirical research using adaptive testing and educational games indicates that success rates in games between 60% and 80% are ideal for maintaining user engagement and discriminating individual differences among players’ gameplay (Embretson & Reise, 2013; Schell, 2008).

In addition, our exploratory data analysis revealed that players ask mostly concrete questions, that the number of questions and complexity characterize overall game performance, and that eliminating suspects by visually “crossing them out” in the game facilitates winning the game. Additionally, we developed measures to capture the effectiveness of the questions asked by players, the question effectiveness measure (QEM). This measure is found to be indicative of successful gameplay.

Considering frameworks such as Optimal Experimental Design, which suggests that questions are formulated to maximize information gain and minimize uncertainty (i.e., entropy), our findings align well with this theory (Raz & Kenett, 2024). Effective questions, indicated by higher QEM scores, were associated with fewer guesses and more efficient gameplay. Ruggeri et al.’s (2017) research on hypothesis-scanning and constraint-seeking questions further supports the idea that strategic question-asking is crucial for problem-solving. In our studies, participants who asked more targeted, constraint-seeking questions tended to perform better, supporting the notion that these questions are more efficient in narrowing down possibilities and identifying the correct suspect. Thus, asking more complex questions at the outset of such games indicates asking better questions that likely facilitate optimal game strategy (Nelson, 2005; Rothe et al., 2018).

While the questions players ask in the game are largely concrete and focus on the physical features of the suspects, the number of questions asked and their complexity impact their gameplay and relate to their cognitive capacities. The number of questions asked was related to their effectiveness, as measured via the QEM. Critically, the complexity of the questions asked in the game, measured via the Bloom taxonomy, was related to intelligence, the effectiveness of the questions, and the number of eliminated suspects (which facilitates winning in the game). Furthermore, our findings indicate that more complex initial questions asked by players can help in eliminating more suspects and lead to fewer overall questions being asked later in the game. Additionally, we found that more complex first questions tend to affect the overall questioning strategy, specifically by being followed by less complex second questions. Thus, our game enables elucidating the contributions of the fluency and complexity of questions asked in such games that require convergent question-asking strategies. Convergent thinking has been strongly related to intelligence and is critical for the evaluation processes taking place during creative thinking (Dechaume et al., 2024; Eymann et al., 2024; Lee & Therriault, 2013). As such, our game allows exploring a critical stage of the creative process (Benedek et al., 2023).

### The relation between game performance and cognitive assessments

Correlations between various cognitive measures and gameplay strategies were observed in an exploratory fashion, such as the relationship between intelligence scores, the effectiveness of the questions (QEM), and the number of suspects eliminated during the gameplay. Moreover, the number of suspects eliminated predicts with high accuracy the game outcome, and was related to higher intelligence, more complex question-asking, and less curiosity. While our study was essentially an exploratory proof of concept, these relations overall replicated across both studies. However, factors other than the sheer number of eliminations, such as the accuracy of the eliminations or other gameplay elements, may also play a critical role and require future examination.

A consistent finding across both studies is the relation between game performance and intelligence. Specifically, higher fluid intelligence (*Gf*) and broad retrieval ability (*Gr*) were strongly linked to more effective questioning strategies, leading to successful outcomes. To delve into this relation, we conducted an exploratory analysis, splitting the sample collected in Study 2 into lower- vs. higher-*Gf* participants. Given the strong correlation between *Gf* and *Gr*, and the exploratory nature of this analysis, we only conducted such an analysis for *Gf*. This analysis reveals how distinct patterns in how intelligence levels influence the relationships between game performance, creativity measures, and questioning strategies. In the lower intelligence group, higher complexity in initial questions was linked to fewer questions. In contrast, the higher intelligence group showed a strong connection between intelligence measures and complex questioning, with curiosity negatively impacting effectiveness in the game. These findings highlight the nuanced connection between cognitive abilities and strategic decision-making in problem-solving scenarios. Yet, such results must be considered as exploratory and require future studies to replicate and generalize these preliminary findings.

Despite our findings relating intelligence and performance in our game exploratory, they align with studies on intelligence in other complex environments, such as the learning strategies of novice online chess players (Kuperwajs et al., 2024). In both Spot the Spy and chess, success hinges on the players’ ability to navigate a landscape of numerous possible actions. While chess operates within a closed system with finite moves, Spot the Spy presents a more open-ended scenario, akin to real-world challenges where the options are wide and less predictable. This research shows that intelligent behavior in complex settings requires adaptive decision-making, where strategies are refined based on experience and observation. Similarly, in our study, participants with higher intelligence and creativity demonstrated a greater ability to craft effective questions, leading to better outcomes. This highlights the deep connection between cognitive abilities and strategic thinking in both structured and unstructured environments (Allen et al., 2024; Pedersen et al., 2023; Raz, Reiter-Palmon, et al., 2024).

### Divergent thinking, question-asking, and game performance

In both studies we measured participants’ performance in the AUT, which is the most widely used task to assess individual differences in divergent thinking (Acar & Runco, 2019; Runco & Acar, 2012); and the AQT, which empirically measures open-ended question-asking and has been related to creativity (Raz et al., 2023; Raz, Reiter-Palmon, et al., 2024; Raz et al., 2025). These were collected to examine our second hypothesis, regarding the role of individual differences in creativity in game performance.

Across both studies, we did not find any significant relation between participants' AUT scores and their performance in the game. This is surprising given the findings of Acar et al. (2023), who show that higher creative individuals (based on AUT scores) tend to ask more open-ended questions and that closed-ended questions were a strong predictor of originality. However, our findings are not surprising given the weak ecological validity of divergent thinking in predicting real-life creativity (Said-Metwaly et al., 2024; Zeng et al., 2011).

Examining the relation between participants’ performance in the AQT and their gameplay resulted in mixed findings. While in Study 1 we did not find any significant relations, Study 2 revealed several relations between AQT and game measures. Specifically, our results showed that participants with higher AQT fluency asked more effective questions in the game—reflecting their ability to approach the problem from multiple angles—as well as eliminating more suspects—which facilitates winning the game. These results echo the findings of Albergaria-Almeida (2011), who noted that higher creativity levels are associated with a greater diversity of questions (see also Acar et al., 2023). Furthermore, the complexity of questions asked by participants in the AQT was positively related to the number of questions they asked in the game. Since the number of questions asked in the game was positively related to game performance, this could reflect a general tendency to ask more complex questions in such a context. However, we did not find any significant correlation between the complexity score in the AQT and its counterpart in the game. Thus, further research is needed to examine such relations.

Recently, Raz, Reiter-Palom, et al. (2024) examined how performance in the AUT and AQT predicted successful solving of open-ended and closed-ended problems. The authors find that while AUT originality predicted closed-ended problem solving, AUT fluency and AQT complexity predicted open-ended problem solving (see Raz, Reiter-Palmon, et al., 2024). In Study 2, when we split the sample into lower- and higher-intelligence groups, we replicated the results of Raz et al., (2024a, 2024b), finding that AUT originality was related to performance in our closed-ended game, as well as replicating the results of our Study 2, i.e., AQT fluency was related to higher effective game performance. These results further highlight the complex relationship between intelligence, creativity, question-asking, and performance in our game, which requires further extensive research.

Given our results and the findings of Raz, Reiter-Palmon, et al. (2024), one crucial question is whether our closed-ended Spot the Spy game is suitable for studying in relation to open-ended cognitive assessments based on the AUT and AQT? First, Spot the Spy is developed to allow empirical study of question-asking in natural environments, and is not explicitly focused on creativity. Its purpose is to advance empirical investigation for researchers interested in studying question-asking, and it can be used in conjunction with any other cognitive assessment. The flexibility of the game allows coding additional variables as well as further developing the game (i.e., more characters, more features, different prompts to the AI agent). Second, partially supporting our second hypothesis, we do find some complex relations between measures of the AUT and AQT in relation to game performance. Indeed, much more work is needed to elucidate the role of such open-ended divergent thinking capacities in real-life contexts, especially due to issues of ecological validity (Said-Metwaly et al., 2024).

### Limitations

Our study entails several limitations. Being exploratory in nature, the research primarily serves as a starting point for more in-depth investigations. The sample sizes, while adequate for preliminary analysis, could be expanded in future studies to enhance the generalizability of the findings. The use of an online game for data collection will be useful for crowdsourcing on a large scale. Additionally, the game environment, though designed to mimic real-life problem-solving scenarios, still possesses inherent limitations of artificial settings. Future research could focus on diversifying the contexts in which question-asking is analyzed, including more varied real-world scenarios and greater complexity of the attributes of the characters. In addition, future studies should implement a wider range of measures, quantitatively analyzing the content of the questions—not just complexity based on the Bloom taxonomy—to quantify additional dimensions of such questions (e.g., Chen et al., 2024).

Furthermore, future studies should explore ways to develop our game to be more open-ended compared to its current closed-ended design. Given that past work has shown how complex question-asking facilitates open-ended and not closed-ended problem solving (Raz, Reiter-Palmon, et al., 2024), such game development is critical to elucidate complex question-asking. This can either be in the Spot the Spy game, i.e., by increasing the ambiguity of the task or adding more degrees of freedom (such as more than one spy or more ambiguous responses from the chatbot agent), or by developing additional question-asking open-ended games.

Another notable limitation is the use of an AI-powered chatbot to provide answers during the game. Some of the answers given by the chatbot were not accurate, which could have impacted the players’ strategies and overall performance. This underscores the importance of effective prompt engineering to ensure that the AI provides accurate and relevant responses. The art of crafting cues for language models, known as prompt engineering, shares commonalities with question-asking in terms of creativity, critical thinking, and the need to be clear and precise. According to a theoretical exploration by Sasson-Lazovsky et al. (2025), prompt engineering and question-asking form a symbiotic relationship, emphasizing the dynamic role of creativity in both processes. Enhancing the AI’s ability to understand and respond accurately to player inquiries is crucial for future iterations of this research, as it would lead to more reliable and valid results.

Finally, our QEM measure has limitations. If a question could not be classified based on the predefined mappings, it was excluded from the sequence. This could potentially omit important questions from the analysis. Additionally, the method did not take into account the actual answer received by the chatbot during the game, focusing solely on the potential suspects eliminated based on the ground truth, while not reflecting the actual number of suspects the player could eliminate based on the information provided. A limitation of the effective QEM metric arises when players ask more questions than necessary, a situation we refer to as "overshooting." Once a player has effectively eliminated all suspects, any further questions may lead to an attempt to divide by zero in the metric's formulation, as the denominator (total number of suspects left) would be zero. This can result in undefined values or negative scores. This limitation underscores the need for careful monitoring of the metric's application, particularly in scenarios where players might continue to ask questions despite already having enough information to identify the target. Thus, further development of such information gain measures, which we quantify here by the QEM and effective QEM, is needed (Nelson, 2005; Ruggeri et al., 2017). Such a measure of development is needed to study how the process of question-asking empirically narrows the information space, and whether it is serial, brute-force, or more parallel in nature.

### Conclusion

In the current study, we develop and present the Spot the Spy game, a question-asking, hidden-object-based online game that allows for empirically investigating question-asking in more ecological settings, based on gaming environments (Allen et al., 2024). After developing the game, we validate it via an online exploratory study, and then replicate these findings with a second in-lab hypothesis-driven study. In both studies, we examine participants’ gameplay alongside the assessment of related cognitive capacities (creativity, intelligence, curiosity). This research represents a step forward in elucidating the role of question-asking in cognitive processes, particularly within the context of academic games. By developing and utilizing the Spot the Spy game, we were able to quantitatively analyze the relationship between question-asking strategies in the game and various cognitive abilities in a natural context, “outside of the lab”.

The findings from our validation and replication studies indicate an intricate connection between intelligence, creativity, and question-asking. The success of Spot the Spy in eliciting meaningful data on question-asking strategies demonstrates the viability of using game-based environments for investigating complex cognitive abilities. The research highlights the significance of strategic question-asking in problem-solving within more ecological settings, such as gaming environments, demonstrating that effective and targeted questions are crucial for success. The positive correlations between cognitive abilities, such as intelligence and creativity, and question-asking effectiveness underscore the role of individual cognitive differences in inquiry. These insights can impact educational practices, encouraging the development of curricula that foster strategic question-asking to enhance learning and critical thinking. Moreover, the use of AI-powered chatbots and interactive systems in the game underscores the potential for improving AI design to support and analyze inquiry-based learning (Sasson-Lazovsky et al., 2025). Future research is needed to further unravel the complexities of question-asking in open-ended, and not just closed-ended, scenarios. Critically, our developed game paves the way for further empirical inquiry, leading to a deeper understanding of the cognitive underpinnings of one of our most fundamental human abilities – asking questions.

## Data Availability

Data and materials are available at https://osf.io/afrbs/ and https://github.com/galsasson1/SpotTheSpyGame

## Code availability

Code is available at https://osf.io/afrbs/
